# Polyphenol Profile and Pharmaceutical Potential of *Quercus* spp. Bark Extracts

**DOI:** 10.3390/plants8110486

**Published:** 2019-11-09

**Authors:** Hosam O. Elansary, Agnieszka Szopa, Paweł Kubica, Halina Ekiert, Mohamed A. Mattar, Mohamed A. Al-Yafrasi, Diaa O. El-Ansary, Tarek K. Zin El-Abedin, Kowiyou Yessoufou

**Affiliations:** 1 Plant Production Department, College of Food and Agriculture Sciences, King Saud University, Riyadh 11451, Saudi Arabia; 2 Floriculture, Ornamental Horticulture, and Garden Design Department, Faculty of Agriculture (El-Shatby), Alexandria University, Alexandria 21526, Egypt; 3 Department of Geography, Environmental Management, and Energy Studies, University of Johannesburg, APK campus, Johannesburg 2092, South Africa; kowiyouy@uj.ac.za; 4 Department of Pharmaceutical Botany, Medical College, Jagiellonian University, ul. Medyczna 9, 30-688 Kraków, Poland; a.szopa@uj.edu.pl (A.S.); p.kubica@uj.edu.pl (P.K.); mfekiert@cyf-kr.edu.pl (H.E.); 5 Department of Agricultural Engineering, College of Food and Agriculture Sciences, King Saud University, Riyadh 11451, Saudi Arabia; mmattar@ksu.edu.sa (M.A.M.); tarekzein137@yahoo.com (T.K.Z.E.-A.); 6 Precision Agriculture Laboratory, Department of Pomology, Faculty of Agriculture (El-Shatby), Alexandria University, Alexandria 21526, Egypt

**Keywords:** *Quercus* spp. bark extract, flavan-3-ols, phenolic acids, antimicrobial, antioxidant, cytotoxicity

## Abstract

Targeted profiling of polyphenols in trees may reveal valuable sources of natural compounds with major applications in pharmacology and disease control. The current study targeted the profiling of polyphenols using HPLC-DAD in *Quercus robur*, *Q. macrocarpa* and *Q. acutissima* bark extracts. Free radical scavenging of each extract was investigated using antioxidant assays. Antimicrobial activities against a wide spectrum of bacteria and fungi were explored, as well as anticancer activities against different cancer cell lines. The HPLC-DAD analyses revealed the availability of several polyphenols in high amounts, including ellagic acid (in *Q. robur*) and caffeic acid (in *Q. macrocarpa*) in all three species. The bioactivity assay revealed high antioxidant activity in *Q. robur* compared to that of the other species, as well as phenolic standards. The three oak bark extracts showed clear antibacterial activities against most bacteria tested, with the highest antibacterial activities in the extracts of *Q. robur*. In addition, the three extracts showed higher antibacterial activities against *Pseudomonas aeruginosa, Micrococcus flavus,* and *Escherichia coli* compared to that of other bacteria. There were strong antifungal activities against some fungi, such as *Aspergillus flavus*, *Penicillium funiculosum,* and *Penicillium ochrochloron*. There were also noticeable anticancer activities against MCF-7, HeLa, Jurkat, and HT-29 cell lines, with the highest anticancer activity in the extracts of *Q. robur*. This is the first study that reveals not only novel sources of important polyphenols (e.g., ellagic acid) in *Q. robur*, *Q. macrocarpa* and *Q. acutissima* bark but also their anticancer activities against diverse cancer cell lines.

## 1. Introduction

Tree barks are widely used in traditional medicine to treat several diseases because of their medicinal properties grounded in the presence of phenolic compounds that can have antioxidant, antimicrobial, and anti-inflammatory activities [1,2,3]. The bark of *Quercus* species in particular is receiving increased attention because of its diverse traditional medicinal uses, its abundance and the low price of its wood residues, such as bark [4]. The genus *Quercus,* belonging to the Fagaceae family, contains trees that are distributed worldwide, with an estimated 450 species [5,6]. There are differences in their morphological appearance and chemical composition.

The best-known species in Europe is *Quercus robur* L. (known as common oak). This plant occurs naturally in Europe, Asia, and North America and is used in traditional medicine for the treatment of diarrhea and inflammation [7]. The bark of *Q. robur* is listed in the official database of pharmaco-therapeutic plants by the European Medicine Agency [8]. The European Pharmacopoeia [9,10] referred to the raw plant material of *Q. robur* as the cut and dried bark of young branches and lateral shoots, which contain a minimal amount of 3% tannins expressed as pyrogallol and calculated with reference to the dried herbal substance. *Q. robur* bark contains a high amount of tannins (hydrolyzable and condensed tannins) (8%–20%). These tannins are composed of either galloyl esters and their derivatives (gallotannins, ellagitannins, and complex tannins) or oligomeric and polymeric proanthocyanidins and can possess different interflavanyl couplings and substitution patterns (condensed tannins) [11,12]. However, there is no information in the literature regarding the phenolic profile of bark extracts of this species. Although it was reported that *Q. robur* barks from Poland have strong antioxidant activities [2], the phenolic composition of these barks and the respective bioactivities of their compounds remain unexplored. 

Furthermore, *Quercus acutissima* Carruth. (sawtooth oak) is another naturally occurring species, native to eastern and southern regions of Asia and naturalized in some eastern regions of North America. The fruits are not preferable as food for cattle because of their poor taste, and the wood is of low quality. However, the use of *Q. acutissima* as a medicinal plant has been mentioned in traditional Asian medicine, especially bark extracts which are used in the treatment of skin disorders, and some studies have confirmed the effectiveness of these extracts in this regard (i.e., in the treatment of skin disorders) [13,14].

As far as the species *Quercus macrocarpa* Michx. (bur oak) is concerned, it is native to North America. This species is included in the Red List Species Program by the International Union for Conservation of Nature and Natural Resources as a species of least concern [15]. *Q. macrocarpa* is a valuable species for cultivation with high drought tolerance [16]. Little is known about the chemical composition and possible bioactivity of its bark. 

Overall, the *Quercus* genus is distributed worldwide, with the traditional use of potentially bioactive raw material in specific regions [8,17]; however, information from experimental studies regarding the bioactivity (e.g., anticancer activity) of the bark is limited. In this study, the polyphenol profile of three *Quercus* spp. (*Q. acutissima, Q. macrocarpa,* and *Q. robur*) was evaluated for the first time by HPLC-DAD analysis. Moreover, the antioxidant, antibacterial, antifungal, and anticancer activities were explored using different antioxidant methods, a wide spectrum of bacteria and fungi as well as different human cancer cell lines. 

## 2. Results

### 2.1. Targeted Profiling of Biologically Active Metabolites 

#### 2.1.1. *Quercus Acutissima*

**Figure 1:**
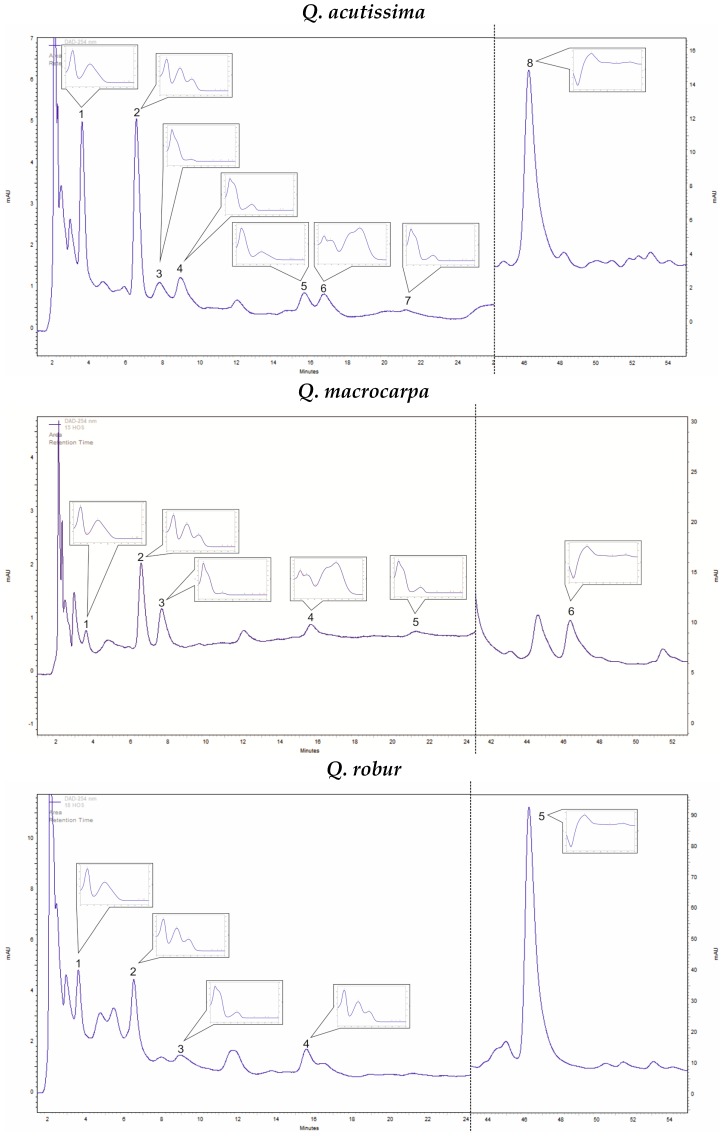
Representative of HPLC-DAD (λ = 254 nm) chromatograms of *Quercus* ssp. bark extracts. ***Q. acutissima;*** 1—Gallic acid, 2—Protocatechuic acid, 3—Epigallocatechin, 4—Catechin, 5—Epigallocatechin gallate, 6—Caffeic acid, 7—Epicatechin, 8—Ellagic acid. ***Q. macrocarpa;*** 1—Gallic acid, 2—Protocatechuic acid, 3—Epigallocatechin, 4—Caffeic acid, 5—Epicatechin, 6—Elagic acid. ***Q. robur;*** 1—Gallic acid, 2—Protocatechuic acid, 3—Catechin, 4—Vanillic acid, 5—Ellagic acid.

In *Q. acutissima* methanolic bark extracts, only four phenolic acids (caffeic acid, ellagic acid, gallic acid, and protocatechuic acid) were confirmed out of the 21 screened (Table 1). The dominant compound was ellagic acid (13.50 mg 100 g^−1^ DW), followed by gallic acid (7.09 mg 100 g^−1^ DW). The amounts of protocatechuic and caffeic acids were lower. Out of the five analyzed catechin derivatives in the bark extracts, four were detected (catechin, epicatechin, epigallocatechin, and epigallocatechin gallate; Table 1). Their amounts were comparable and ranged from 8.31 to 12.91 mg 100 g^−1^ DW. Quantitatively the dominant compounds were epicatechin (12.66 mg 100 g^−1^ DW) and epigallocatechin (12.66 mg 100 g^−1^ DW) (Figure 1). 

#### 2.1.2. *Quercus Macrocarpa*

In *Q. macrocarpa* bark extracts, a very high amount of caffeic acid was detected at 100.58 mg 100 g^−1^ DW (Table 1). Other phenolic acids, including ellagic, protocatechuic, and gallic acid, were also confirmed, but in much lower amounts of 5.07, 3.36, and 0.87 mg 100 g^−1^ DW, respectively. Two catechin derivatives were found: epicatechin – 11.00 mg 100 g^−1^ DW and epigallocatechin – 10.15 mg 100 g^−1^ DW (Table 1 and Figure 1). 

#### 2.1.3. *Quercus Robur*

In *Q. robur* bark extracts, four phenolic acids (out of 21 compounds) were detected: ellagic acid, gallic acid, protocatechuic acid, and vanillic acid. The dominant compound was ellagic acid (97.82 mg 100 g^−1^ DW), whereas the amounts of the other compounds were lower (gallic acid—8.23 mg 100 g^−1^ DW, protocatechuic acid—6.96 mg 100 g^−1^ DW, and vanillic acid—2.61 mg 100 g^−1^ DW). In the studied extracts, a high amount of catechin was estimated at 44.52 mg 100 g^−1^ DW (Table 1 and Figure 1). 

### 2.2. *Antioxidant Activities*

The antioxidant activities of the bark extracts of the three species as well as ellagic and caffeic acids are shown in Table 2. *Q. robur* showed significantly higher antioxidant activities by means of the DPPH (IC_50_, 3.0 µg mL^−1^), β-carotene bleaching (IC_50_, 3.3 µg mL^−1^), FRAP (IC_50_, 3.8 mM TEAC g^−1^ extract) assays compared to other species. *Q. macrocarpa* exhibited higher antioxidant activities than *Q. acutissima*. Furthermore, *Q. robur* antioxidant activities were comparable to standard antioxidants (BHT). The antioxidant activities of ellagic and caffeic acids were comparable to those of *Q. robur* and *Q. macrocarpa*, respectively. 

### 2.3. Antibacterial Activities 

The antibacterial activities of the bark extracts of *Q. acutissima, Q. macrocarpa, Q. robur* as well as ellagic and caffeic acids using the micro-dilution methods are shown in Table 3. The three extracts exhibited clear antibacterial activities against most species of microorganism studied. The MIC values ranged between 0.04 and 0.29 mg mL^−1^, whereas the MBC ranged between 0.11 and 0.66 mg mL^−1^. The response of the bacterial species to the extracts used varied among species. The highest antibacterial activities were found for the extracts of *Q. robur* compared to those of the other two species. The three extracts exhibited higher antibacterial activities against *Pseudomonas aeruginosa*, *M. flavus* and *E. coli* compared to other bacterial species. Further, their antibacterial activities were comparable to those of antibiotics. The antibacterial activities of phenolic standards of the ellagic and caffeic acids were comparable and higher than those of *Q. robur* and *Q. macrocarpa* extracts, respectively. 

### 2.4. Antifungal Activities

The extracts were screened for their antifungal activities against several fungi, as shown in Table 4. The MIC ranged between 0.16 and 2 mg mL^−1^, whereas the MFC ranged between 0.23 and 3.61 mg mL^−1^. There were obvious antifungal activities against some fungi, including *A. flavus*, *Penicillium funiculosum,* and *Penicillium ochrochloron*. However, *A. ochraceus* and *A. niger,* as well as *C. albicans,* showed slight resistance to the extracts. The activities of the extracts were comparable to commercial reagents in most cases. The antifungal activities of phenolic standards of the ellagic and caffeic acids were comparable to those of *Q. robur* and *Q. macrocarpa* extracts, respectively. 

### 2.5. Anticancer Activities

**Figure 2:**
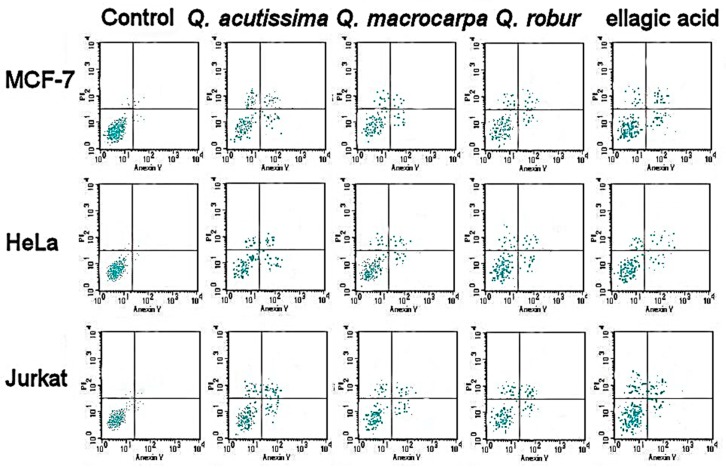
Apoptotic cell population (IC_30_) using flow cytometry.

**Figure 3:**
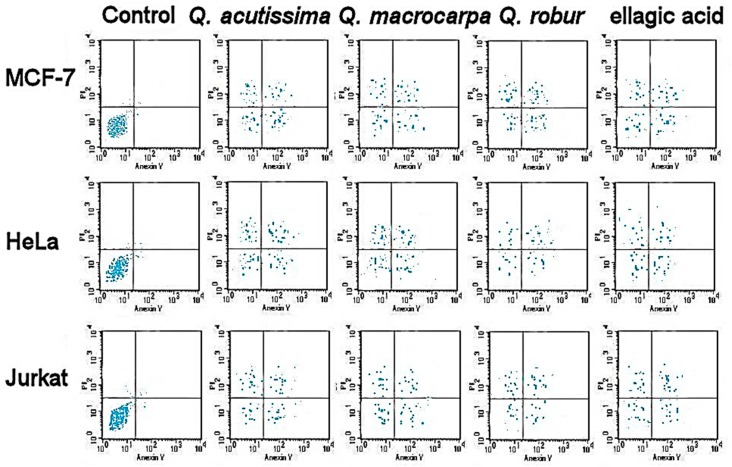
Apoptotic cell population (IC_50_) using flow cytometry.

The bark extracts were screened for their anticancer activities against different cancer cell lines, as shown in Table 5. There were obvious anticancer activities against MCF-7, HeLa, Jurkat, and HT-29 cell lines. The highest anticancer activity was found in the extracts of *Q. robur* compared to that of *Q. macrocarpa* and *Q. acutissima*. Only *Q. robur* exhibited anticancer activity against T24. The anticancer activities of phenolic standards of the ellagic and caffeic acids were comparable to those of *Q. robur* and *Q. macrocarpa* extracts, respectively. The apoptotic assay revealed the accumulation of necrotic as well as both early and late apoptotic cells in different treatments in a dose dependent manner (Figure 2 and Figure 3).

## 3. Discussion

The HPLC-DAD analyses of methanolic extracts of the bark of three *Quercus* species indicated that specific phenolic acids and catechin derivatives were the major active ingredients. Three phenolic acids were common in all three bark extracts (ellagic acid, gallic acid, and protocatechuic acid) and they are benzoic acid derivatives. Ellagic acid and gallic acid are known derivatives produced by tannin hydrolyses, typical for the *Quercus* species [2,12]. 

Interestingly, extremely high amounts of ellagic acid were found in *Q. robur* bark extract (97.82 mg 100 g^−1^ DW) that were 7-fold that of *Q. acutissima* and 17-fold that of *Q. macrocarpa* bark extracts (Table 1). In *Q. acutissima* and *Q. macrocarpa* bark extracts, there were noticeable amounts of caffeic acid. In *Q. macrocarpa,* the caffeic acid content was −100.58 mg 100 g^−1^ DW (23-fold that of *Q. acutissima*) (Table 1). In *Q. robur* bark extract, vanillic acid was detected (Table 1). This phenolic acid was not detected in other *Quercus* species. In the *Quercus* species, the most often studied bioactive metabolite content is that of leaf and needle extracts of *Q. robur* [18]. A previous study documented some phenolic acids, including *p*-hydroxybenzoic acid, vanillic acid, gallic acid, syringic acid, ferulic acid, and *o*- and *p*-coumaric acids. For *Q. acutissima*, gentisic acid (phenolic acid) was confirmed in the extracts of fresh acorns [19]. However, in the available literature, no information regarding phenolic acid estimation in *Q. macrocarpa* or in the bark extracts of the three *Quercus* species was found. The latter is important because the cortex is recognized as the raw material of oaks. This study demonstrated differences in secondary metabolite composition among the examined cortex extracts and is the first to document the phenolic acid profiles in these materials. 

The detected phenolic acids in the studied extracts are very important from a pharmacological and economic point of view. For example, gallic acid has antibacterial, hypoglycemic, anticancer, and antimutagenic activities [20,21]. In agreement with the current study, ellagic acid is known for strong antioxidative, antiproliferative, and anticancer properties [22,23]. Protocatechuic acid has antifungal, antibacterial, antiviral, anti-inflammatory, antiatherosclerotic, antiulcer, and anticancer properties [20,24,25]. Vanillic acid also exhibits antioxidant and hepatoprotective actions [26,27]. The presence of these compounds, in addition to tannins, contributes to the pharmacological activities of these raw materials.

The *Quercus* cortex is recognized in phototherapy as a valuable plant raw material because of its extremely high tannin content [10]. In the current study, the chromatographic analyses detected catechin and some derivatives, epicatechin, epigallocatechin, and epigallocatechin gallate, in bark extracts (Table 1). The presence of these compounds was confirmed in the *Q. robur* bark extract [28]. However, little is known about secondary metabolites polyphenolic compositions in the bark extracts of *Q. acutissima* and *Q. macrocarpa.* Only the presence of catechin was previously described in *Q. acutissima* [13,19]. Our study has contributed to the greater understanding of the tannin composition of *Q. acutissima* and *Q. macrocarpa.*

Q. *robur* exhibited significantly higher antioxidant activities by means of the DPPH and β-carotene bleaching assays compared to that of the other species and had activities comparable to that of standard antioxidants. Such important antioxidant activities are primarily attributable to the major bioactive compound, which is ellagic acid. High antioxidant activities were described for ellagic acid [29]. In agreement with our results, a study [2] reported strong antioxidant activities in *Q. robur* bark from Poland; however, they did not detect the phenolic profile of those trees. A recent investigation on *Q. robur* and Q. *petraea* leaves, twigs, and acorns from Serbia revealed strong antioxidant activities [5]. *Q. macrocarpa* had higher antioxidant activities than *Q. acutissima* and this can be explained by the extremely high amount of caffeic acid in *Q. macrocarpa*. Caffeic acid is known for antioxidant, antibacterial, and antifungal activities [30], which is in agreement with the results of the current study.

The three extracts showed antibacterial activities against most bacteria species studied and the highest antibacterial activities were found in the extracts of *Q. robur* as compared to that of the other two species. A previous report on *Q. robur* from Finland documented some antibacterial activity of the bark extract on *S. aureus* and *C. albicans* using the agar diffusion method [31]. In the current study, strong antibacterial activities were found against *Pseudomonas aeruginosa, M. flavus,* and *E. coli* and moderate activities against other bacterial species. The work on *Q. robur* bark bioactivities is relatively limited, but other species, such as *Q. cortex,* have revealed some antibacterial activities against *Chromobacterium violaceum* [32]. These strong antibacterial activities might be attributed to ellagic acid, which has some antibacterial activities against certain bacteria, such as *Streptococcus mutans*, *Streptococcus sanguis,* and *Streptococcus salivarius* [33]. Additionally, the catechin-rich sources revealed in this study have obvious strong antibacterial and antifungal activities and are comparable to other genera. Green tea (*Camellia sinensis*) are flavan-3-ols that have moderate to strong antibacterial activities against Gram-positive and Gram-negative bacterial species [34]. Furthermore, green tea polyphenols have been associated with some antifungal effects against *C. albicans* [35]. In the current study, obvious antifungal activities were found against *A. flavus*, *Penicillium funiculosum,* and *Penicillium ochrochloron,* as well as moderate activities against *C. albicans*. Such effects are mainly attributable to specific polyphenols such as ellagic acid, which are flavan-3-ols, and caffeic acid. These polyphenols might be the major component of the raw material as in the bark of oaks. 

There were anticancer activities against MCF-7, HeLa, Jurkat, and HT-29 cell lines and the highest activities were found in the extracts of *Q. robur* compared to that of *Q. macrocarpa* and *Q. acutissima*. A previous report documented that *Q. petraea* (stem bark) and *Q. robur* (leaf) extracts have potent inhibitory activities against LoVo colon, PC3 prostate, and U373 glioblastoma cancer cell lines, but they did not describe the active ingredients in the extracts [36]. Furthermore, the use of *Q. robur*, *Q. macrocarpa,* and *Q. acutissima* bark extract as an anticancer agent is novel. The anticancer activities are mainly attributed to major constituents, such as the ellagic acid found in this study. Also, flavan-3-ols have previously shown anticancer activities [37]. The oak barks selected for the current study are valuable sources of anticancer, antioxidant, and antimicrobial natural compounds.

## 4. Materials and Methods 

### 4.1. Plant Material and Sample Preparation

The outer bark of *Q. acutissima, Q. macrocarpa,* and *Q. robur* (Fagaceae family) were sampled from the University of Guelph Arboretum in Guelph, Ontario, Canada, identified by Hosam Elansary, and then vouchered at the University of Guelph and at Alexandria University (Hosam000975–2018). The *Quercus* spp. bark samples were dried by lyophilization (Labconco, USA) and then powdered. The dried pulverized plant samples of 0.5 g DW (dry weight) each, in 3 replications, were put in 15 mL tubes and subjected to extraction with 10 mL methanol (Chempur, Poland) by sonication (2 × 30 min at 30 °C) in an ultrasonic bath (Sonic-2, POLSONIC; ultrasonic power 2 × 100W, 40 kHz, water bath dimensions 150 × 135 × 100 mm). The extracts were filtered using Whatman paper and left in crystallizers to evaporate methanol at room temperature. The dry residue was dissolved in 2 mL of methanol (Merck, HPLC grade purity) [38]. Then the samples were stored at −80 °C for future bioassays. For bioassays, the methanol was totally removed by evaporating the methanol in a rotary evaporator. Analytical/HPLC grade chemicals were used (Sigma Aldrich, Germany) for the bioassays. The bacterial and fungal cultures were obtained from the Department of Floriculture and Ornamental Horticulture, Faculty of Agriculture, Alexandria, Egypt. Cell cultures of breast adenocarcinoma (MCF-7), cervical adenocarcinoma (HeLa), T-cell lymphoblast like (Jurkat), colon adenocarcinoma (HT-29), urinary bladder carcinoma (T24), and HEK-293 (human normal cells) were purchased from American Type Culture Collection (ATCC). 

### 4.2. Chemicals

The following standards were used for phenolic acid quantification: benzoic acid and its derivatives (3,4-dihydroxyphenylacetic, ellagic, gallic, gentisic, *p*-hydroxybenzoic, protocatechuic, salicylic, syringic, and vanillic acids); cinnamic acid and its derivatives (caffeic, *o*-coumaric, *m*-coumaric, *p*-coumaric, ferulic, hydrocaffeic, isoferulic, and sinapic acids); and depsides (chlorogenic, neochlorogenic, and rosmarinic acids). To quantify the flavonoids, aglycone (kaempferol, luteolin, myricetin, quercetin and rhamnetin) and glycoside (apigetrin, cynaroside, hyperoside, isoquercetin, quercitrin, robinin, rutin, trifolin, vitexin) standards were used. To quantify the catechin derivatives, epicatechin, epicatechin gallate, epigallocatechin, epigallocatechin gallate and catechin were used. All the substances were acquired from Sigma-Aldrich, Germany.

### 4.3. Analyses of Phenolic Compounds

Analyses of the bark methanolic extracts were performed by a HPLC method [39,40] using the Merck-Hitachi liquid chromatograph (LaChrom Elite) with a DAD detector L-2455. The Purospher RP-18e (250 × 4 mm; 5 μm, Merck) column was used and the temperature was set to 25 °C. The mobile phase consisted of A—methanol, B—methanol: 0.5% acetic acid 1:4 (*v/v*). The flow rate was 1 mL/min, the gradient was as follows: 100% B for 0–20 min; 100–80% B for 20–35 min; 80–60% B for 35–55 min; 60–0% B for 55–70 min; 0% B for 70–75 min; 0–100% B for 75–80 min; 100% B for 80–90 min. The injection volume was 20 µL and the compounds of interest were detected at 254 nm. The applied HPLC method was previously validated by our group [39]. The tested parameters were the following: accuracy; precision at three levels of standard substance concentrations in solution, 50%, 100%, and 150%; linearity; limit of detection (LOD); and limit of quantification (LOQ) [39]. Identification of compounds was performed either by comparison with UV spectra and retention times of reference substances or using co-chromatography (Figure 1). The compounds were quantified using the calibration curves method [38,40,41].

### 4.4. Antioxidant Activity 

DPPH, β-carotene bleaching [42] and ferric reducing antioxidant power (FRAP) [43] assays were used to determine the antioxidant activities of the bark extracts. For DPPH, the samples were incubated for 30 min, and then a wavelength of 517 nm was used to measure absorbance. During the β-carotene bleaching assay, the wavelength of 470 nm was used to determine the absorbance. The amount of the sample (IC_50_ in µg/mL) that scavenged 50% of the DPPH/ β-carotene bleaching solutions was determined by plotting the inhibition percent against extract concentration. A standard antioxidant was used (butylated hydroxytoluene, BHT) as a positive control and the inhibition concentration of each sample was compared with that of the BHT and blank. The FRAP reagent was prepared as described in previous studies (e.g., [43]) using TPTZ (tripyridyl triazine, Sigma-Aldrich, Berlin, Germany). Aliquots (100 μL) of bark extracts or Trolox (Sigma-Aldrich, Berlin, Germany) were added to FRAP reagent (3 mL), mixed, incubated for half an hour at 37 °C and the absorbance was measured at 593 nm. Aqueous solutions of known serial concentrations of Trolox (0–0.5 Mmol/L) were used for the calibration. Two sets of triplicate replications were conducted for all experiments.

### 4.5. Antibacterial Activity

Antibacterial activities of bark extracts were screened against *Bacillus cereus* (ATCC 14579), *Escherichia coli* (ATCC 35210), *Listeria monocytogenes* (clinical isolate)*, Micrococcus flavus* (ATCC 10240), *Pseudomonas aeruginosa* (ATCC 27853), and *Staphylococcus aureus* (ATCC 6538) using the micro-dilution method [44]. Microtiter plates (96-well) containing a serial concentration of bark extract in each well mixed with bacterial inoculum (1.0 × 10^4^ CFU per well) in 100 μL tryptic soy broth were incubated at 37 °C for 24 h in a rotary shaker. The minimum inhibitory concentration (MIC) was defined as the lowest concentration of plant extract that exhibited no visible growth using a binocular microscope and was determined following the incubation period of the microtiter plates. The minimum bactericide concentration (MBC), which was defined as the lowest concentration that caused no visible growth and indicated the killing of 99.5% of the inoculum, was determined using serial subculturing of bark extracts (2 μL). A wavelength of 655 nm was used to determine the optical density in a spectrophotometer. A positive control was used (streptomycin, 0.01–10 mg/mL), as well as a negative one (DMSO, 1%).

### 4.6. Antifungal Activity 

The antifungal activities of bark extracts were determined using a variety of infectious and economically important fungi, including *Aspergillus flavus* (ATCC 9643), *A. ochraceus* (ATCC 12066), *A. niger* (ATCC 6275), *Candida albicans* (ATCC 12066), *Penicillium ochrochloron* (ATCC 48663), and *Penicillium funiculosum* (ATCC 56755). The microdilution method was employed in this assay [45] using bark extract (2 μL) mixed with broth malt medium and the fugal inoculum (spore suspension concentration of 1.0 × 10^5^) in microtiter plates. The plates were incubated at 28 °C for 72 h in a rotary shaker; then the MIC was determined as the lowest concentration inhibiting fungal growth at the binocular microscopic level. The minimum fungicidal concentration (MFC) was defined as the minimum concentration showing no visible growth and indicating the killing of 99.5% of the original inoculum. MFC was determined using serial sub-cultivations of the bark extracts (2 µL) added to 100 µL of broth and inoculum, and then incubated at 28 °C for 72 h. A positive control was used (ketoconazole, 1–3500 µg/mL).

### 4.7. Anticancer Activities 

Cytotoxic activities of the bark extracts were tested on MCF-7, HeLa, Jurkat, HT-29, and T24, as well as HEK-293 (human normal cells) following the MTT method [46]. Briefly, cells were grown in 75 cm^2^ flasks in MEM with 10% FBS, 17.8 mM NaHCO_3_, 0.1 mM non-essential amino acids, and 1 mM sodium pyruvate. They were seeded into 96-well plates at a density of 4 × 10^−4^ per well, left overnight in 270 µL medium, and incubated at 37 °C, 5% CO_2_. Steri-filtered bark extracts were added to the culture media in microtiter plates. Five doses of bark extract were used to reach a final concentration of 50, 100, 200, 300, and 400 µg/mL culture media. Samples were solubilized in DMSO (1%). Untreated cells were considered negative controls and vinblastine sulfate and taxol were used as positive controls. After the incubation of the culture media for 2 days at 37 °C and 5% CO_2_, PBS washing was performed to remove traces of the extract and the medium was supplied by 12 mM MTT dissolved in PBS. Dissolved in isoprobanol, 0.04 N HCl was mixed in each well, allowed to sit for 40 min, and the absorbance was determined at a 570 nm wavelength using a microplate reader (Thermo, MA, USA). The percentage of activity inhibition was calculated in triplicate: 

% Inhibition = (Abs. 570 nm control‒Abs. 570 nm sample)/Abs. 570 nm control × 100. Furthermore, IC_50_ values were obtained by plotting the percentage of cell viability against extract concentration and expressed in µg/mL. The IC_30_ and IC_50_ were used to show the dose dependent apoptotic cell population using flow cytometry (FAC Scan, Becton Dickinson, Iowa, USA) following [46,47,48].

### 4.8. Statistical Analyses 

The least significance difference (LSD) was determined using SPSS software (version 22.0). The quantitative results of chromatographic analyses were expressed in mg 100 g^−1^ dry weight (DW) as the mean ± standard deviation (SD) of three series of experiments.

## 5. Conclusions

This is the first study to profile polyphenols in *Q. robur*, *Q. macrocarpa,* and *Q. acutissima* bark extracts, as well as their bioactivities as antioxidants, antibacterial, antifungal, and anticancer materials. The study revealed the availability of several polyphenols in the three species. The bioactivity assay revealed high antioxidant activity in *Q. robur* compared to that of the other species. The three oak bark extracts showed clear antibacterial activities against most bacteria tested. The highest antibacterial activities were found in the extracts of *Q. robur* and the three extracts showed higher antibacterial activities against *Pseudomonas aeruginosa, M. flavus,* and *E. coli* compared to activities against other bacteria. There were strong antifungal activities against some fungi, such as *A. flavus*, *Penicillium funiculosum,* and *Penicillium ochrochloron*. There were anticancer activities against MCF-7, HeLa, Jurkat, and HT-29 cell lines. The highest anticancer activity was found in the extracts of the *Q. robur* compared to that of *Q. macrocarpa* and *Q. acutissima*. The use of *Q. robur*, *Q. macrocarpa,* and *Q. acutissima* bark extract as an anticancer agent is novel and is attributed to specific phenols such as ellagic acid. The oak bark used in this study are valuable sources of antioxidant, antimicrobial, and anticancer compounds.

## Figures and Tables

**Table 1:** The phenolic acids and catechin derivatives compositions of *Q. acutissima*, *Q. macrocarpa* and *Q. robur* outer bark extracts.

*Quercus Species*	Compound	t_R_	λ_max_	Amount [mg 100 g^−1^] DW
***Q. acutissima***	Catechin	8.96	214, 278	10.52 ± 1.87
	Caffeic acid	16.71	218, 236, 323	4.30 ± 0.05
	Ellagic acid	46.22	253	13.50 ± 2.84
	Epicatechin	21.15	213, 278	12.66 ± 2.97
	Epigallocatechin	7.80	214	12.91 ± 1.91
	Epigallocatechin gallate	15.63	215, 274	8.31 ± 0.03
	Gallic acid	3.61	220, 271	7.09 ± 0.59
	Protocatechuic acid	6.55	220, 259, 294	5.39 ± 0.76
***Q. macrocarpa***	Caffeic acid	15.61	218, 236, 323	100.58 ± 18.02
	Ellagic acid	46.18	253	5.07 ± 0.05
	Epicatechin	21.32	213, 278	11.00 ± 0.34
	Epigalloctechin	7.90	214	10.15 ± 0.32
	Gallic acid	3.58	220, 271	0.87 ± 0.03
	Protocatechuic acid	6.54	220, 259, 294	3.36 ± 0.02
***Q. robur***	Catechin	8.95	214, 278	44.52 ± 5.64
	Ellagic acid	46.22	253	97.82 ± 1.74
	Gallic acid	3.59	220, 271	8.23 ± 0.39
	Protocatechuic acid	6.51	220, 259, 294	6.96 ± 1.14
	Vanillic acid	15.59	219, 260, 293	2.61 ± 0.15

**Table 2:** DPPH and β-carotene bleaching acid of *Q. acutissima*, *Q. macrocarpa* and *Q. robur* outer bark extracts as well as phenol standards. Values are expressed as mean of triplicate determinations ± SD.

	DPPH Free Radical Scavenging Activity (IC_50_, µg mL^−1^)	β-Carotene-linoleic Acid Assay (IC_50_, µg mL^−1^)	FRAP (IC_50_, mM TEAC/g extract)
***Q. acutissima***	4.5 ± 0.1a	4.9 ± 0.1a	5.4 ± 0.1a
***Q. macrocarpa***	3.7 ± 0.1b	4.1 ± 0.1b	4.5 ± 0.1b
***Q. robur***	3.0 ± 0.1c	3.3 ± 0.1c	3.8 ± 0.1d
**ellagic acid**	3.0 ± 0.1c	3.4 ± 0.1c	3.7 ± 0.1d
**caffeic acid**	3.2 ± 0.1c	3.7 ± 0.1c	4.1 ± 0.1c
**BHT**	2.9 ± 0.1c	3.2 ± 0.1c	-
**Trolox**	-	-	3.5 ± 0.1e

Values with different letters within a column indicates significant differences (*p* = 0.05). TEAC: Trolox equivalents antioxidant.

**Table 3:** Minimum inhibitory (MIC) and bactericidal concentration (MBC) of *Q. acutissima, Q. macrocarpa and Q. robur* outer bark extracts (mg mL^−1^) as well as phenolic standards.

	*P. aeruginosa* (ATCC 27853) MIC MBC	*B. cereus* (ATCC 14579) MIC MBC	*L. monocytogenes* (Clinical Isolate) MIC MBC	*E. coli* (ATCC 35210) MIC MBC	*M. flavus* (ATCC 10240) MIC MBC	*S. aureus* (ATCC 6538) MIC MBC
***Q. acutissima***	0.09 ± 0.01	0.17 ± 0.01	0.27 ± 0.02	0.17 ± 0.01	0.17 ± 0.01	0.23 ± 0.01
	0.18 ± 0.02	0.37 ± 0.03	0.66 ± 0.03	0.32 ± 0.02	0.41 ± 0.03	0.46 ± 0.01
***Q. macrocarpa***	0.07 ± 0.01	0.16 ± 0.01	0.29 ± 0.01	0.13 ± 0.01	0.14 ± 0.01	0.22 ± 0.01
	0.15 ± 0.01	0.35 ± 0.03	0.62 ± 0.02	0.29 ± 0.02	0.34 ± 0.03	0.44 ± 0.02
***Q. robur***	0.05 ± 0.01	0.11 ± 0.01	0.25 ± 0.01	0.10 ± 0.01	0.10 ± 0.01	0.23 ± 0.02
	0.11 ± 0.01	0.27 ± 0.02	0.53 ± 0.03	0.21 ± 0.02	0.20 ± 0.02	0.45 ± 0.01
**ellagic acid**	0.04 ± 0.01	0.09 ± 0.01	0.23 ± 0.01	0.09 ± 0.01	0.09 ± 0.01	0.20 ± 0.01
	0.10 ± 0.01	0.22 ± 0.01	0.49 ± 0.02	0.19 ± 0.03	0.18 ± 0.01	0.41 ± 0.03
**caffeic acid**	0.06 ± 0.01	0.13 ± 0.01	0.27 ± 0.01	0.11 ± 0.01	0.13 ± 0.01	0.20 ± 0.01
	0.13 ± 0.01	0.29 ± 0.01	0.58 ± 0.03	0.25 ± 0.01	0.30 ± 0.02	0.41 ± 0.03
**Streptomycin**	0.08 ± 0.01	0.07 ± 0.03	0.14 ± 0.01	0.12 ± 0.01	0.11 ± 0.01	0.19 ± 0.01
	0.16 ± 0.01	0.15 ± 0.01	0.29 ± 0.03	0.27 ± 0.01	0.21 ± 0.02	0.32 ± 0.01

**Table 4:** Minimum inhibitory (MIC) and fungicidal concentration (MFC) of *Q. acutissima, Q. macrocarpa and Q. robur* outer bark extracts (mg mL^−1^) as well as phenolic standards.

	*Aspergillus flavus* MIC MFC	*Aspergillus ochraceus* MIC MFC	*Aspergillus niger* MIC MFC	*Candida albicans* MIC MFC	*Penicillium funiculosum* MIC MFC	*Penicillium ochrochloron* MIC MFC
***Q. acutissima***	0.24 ± 0.01	0.26 ± 0.02	0.21 ± 0.01	0.40 ± 0.02	0.38 ± 0.02	0.25 ± 0.01
	0.51 ± 0.03	0.57 ± 0.02	0.41 ± 0.02	0.86 ± 0.03	0.69 ± 0.03	0.52 ± 0.02
***Q. macrocarpa***	0.22 ± 0.02	0.24 ± 0.03	0.21 ± 0.01	0.34 ± 0.03	0.29 ± 0.03	0.21 ± 0.02
	0.43 ± 0.01	0.48 ± 0.02	0.40 ± 0.03	0.76 ± 0.03	0.68 ± 0.03	0.43 ± 0.03
***Q. robur***	0.19 ± 0.02	0.26 ± 0.01	0.16 ± 0.01	0.31 ± 0.01	0.26 ± 0.01	0.16 ± 0.01
	0.40 ± 0.02	0.53 ± 0.03	0.35 ± 0.02	0.62 ± 0.03	0.63 ± 0.03	0.33 ± 0.03
**ellagic acid**	0.15 ± 0.01	0.22 ± 0.03	0.13 ± 0.01	0.30 ± 0.03	0.23 ± 0.02	0.12 ± 0.01
	0.33 ± 0.03	0.45 ± 0.03	0.28 ± 0.01	0.61 ± 0.03	0.51 ± 0.03	0.25 ± 0.01
**caffeic acid**	0.20 ± 0.01	0.22 ± 0.01	0.20 ± 0.01	0.32 ± 0.01	0.27 ± 0.01	0.20 ± 0.03
	0.40 ± 0.01	0.45 ± 0.01	0.38 ± 0.01	0.64 ± 0.03	0.62 ± 0.02	0.42 ± 0.01
**KTZ**	0.21 ± 0.01	0.21 ± 0.01	0.12 ± 0.01	0.20 ± 0.01	2.00 ± 0.10	0.21 ± 0.01
	0.41 ± 0.01	0.42 ± 0.02	0.23 ± 0.01	0.42 ± 0.01	3.61 ± 0.03	0.42 ± 0.01

**Table 5:** In vitro antiproliferative activity [IC_50_ (µg/mL)] of *Q. acutissima*, *Q. macrocarpa* and *Q. robur* outer bark extracts as well as phenolic standards on cancer cell lines.

	MCF-7	HeLa	Jurkat	HT-29	T24	HEK-293
***Q. acutissima***	52.14 ± 2.1	62.4 ± 2.3	46.2 ± 2.3	173.11 ± 6.7	˃400	˃400
***Q. macrocarpa***	43.54 ± 1.3	54.1 ± 2.1	42.5 ± 1.2	149.24 ± 3.7	˃400	˃400
***Q. robur***	22.10 ± 1.2	31.42 ± 1.0	28.4 ± 2.7	99.8 ± 2.1	290.28	˃400
**ellagic acid**	20.23 ± 1.0	29.33 ± 1.3	27.1 ± 1.6	94.5 ± 1.9	273.31	˃400
**caffeic acid**	40.31 ± 1.9	50.5 ± 2.8	38.85 ± 1.8	131.32 ± 4.1	˃400	˃400
**Vinblastine sulfate**	‒	2.6 ± 0.08	0.1 ± 0.07	21.0 ± 0.5	65.12 ± 3.1	51.4 ± 2.5
**Taxol**	0.09 ± 0.008	‒	‒	‒	‒	‒

## Abbreviations

HPLC-DAD	High-Performance *Liquid Chromatography* with Diode-Array Detection
*Q. acutissima*	*Quercus acutissim*a
*Q. macrocarpa*	*Quercus macrocarpa*
*Q. robur*	*Quercus robur*;
MCF-7	cell cultures of breast adenocarcinoma;
HeLa	cell cultures of cervical adenocarcinoma;
Jurkat	cell cultures of T-cell lymphoblast like;
HT-29	cell cultures of colon adenocarcinoma
T24	urinary bladder carcinoma;
ATCC	American Type Culture Collection;
BHT	butylated hydroxytoluene
DPPH	2,2-Diphenyl-1-picrylhydrazyl
